# Complete Genome Sequence of *Actinoplanes* sp. Strain L3-i22, Isolated from Soil in Japan

**DOI:** 10.1128/MRA.00798-21

**Published:** 2021-10-21

**Authors:** Yoshi Yamano, Sachiko Sugimoto, Katsuyoshi Matsunami

**Affiliations:** a Department of Pharmacognosy, Graduate School of Biomedical and Health Sciences, Hiroshima University, Hiroshima, Japan; SIPBS, University of Strathclyde

## Abstract

Here, we described the closed complete genome sequence of *Actinoplanes* sp. strain L3-i22, which was obtained from the assembly with long reads and subsequent polishing with short reads. The complete genome consists of a 12,014,766-bp chromosome, with a GC content of 71.4%, and contains no plasmids.

## ANNOUNCEMENT

*Actinoplanes* species are Gram-positive filamentous bacteria belonging to the *Micromonosporaceae* family of actinomycetes, which produce various antibiotics, enzymes, and other biologically active substances, and their genomes are a source of secondary metabolites and enzymes (1–5). Here, we present the complete genome sequence of *Actinoplanes* sp. strain L3-i22, which was isolated from soil collected from the ruins of Nissyoen in Hiroshima, Japan (34°25′N, 132°26′E), in December 2020.

First, the soil was suspended in sterile distilled water and filtered with filter paper (filter size, 6 μm; Advantech) to obtain a microbial solution. Next, the microbial solution was diluted in molten SMS medium (0.125 g casein, 0.1 g potato starch, 1 g Casamino Acids, 20 g Bacto agar in 1 liter of water), and an average of one cell per culture pore was fixed in a device (originally created in our laboratory) that has the ability to absorb nutrients from the external environment by cultivating a culture cell between semipermeable membranes, similar to the ichip developed by Nichols et al. (6). The device was then assembled and incubated for 3 weeks in the soil. SMS agar in each well was subsequently dropped into Mueller-Hinton (MH) agar and cultured for 1 month to obtain the colony.

To yield the genomic DNA, strain L3-i22 was cultured at 25°C for 1 week in MH broth, and high-molecular-weight genomic DNA was extracted using a phenol-chloroform extraction technique (7). The genome obtained was subjected to short- and long-read sequencing at the Oral Microbiome Center, Taniguchi Dental Clinic (Japan).

The short-read sequencing library was prepared using the MGIEasy FS PCR-free DNA library preparation kit (MGI Tech Co., Shenzhen, China), according to the manufacturer’s instructions. Paired-end sequencing (150-bp reads) was performed using a DNBseq-G400RS instrument, generating 4,832,110 paired-end reads (1.5 Gb). Next, a DNA library was prepared for long-read sequencing using the ligation sequencing kit (Oxford Nanopore Technologies [ONT]). The prepared library was applied to a FLO-MIN106 R9.41 flow cell (ONT). Furthermore, the long-read sequences, which were base called using Guppy v.4.2.3 in high-accuracy mode, generated 719,321 reads (3.1 Gb), with an average length of 4,092 bp, during a 24-h runtime. Low-quality short reads were trimmed using fastp v.0.20.1 (8), and low-quality Nanopore reads with average Q values of <10.0 were trimmed using NanoFilt v.2.7.1 (9). Next, Flye v.2.8.3 (10) was used for *de novo* genome assembly, followed by polishing using Pilon v.1.23 (11) until no corrections were made, generating a single circular contig for the chromosome. Finally, this sequence was checked for errors derived from misassemblies, including small gaps and misassembly breakpoints, using SV Quest v.1.0 to scan for small and large gaps in the assembly (12). Positions at which assembly errors were identified were manually corrected using sequencing data. Default parameters were used for all software unless otherwise specified.

The chromosome of *Actinoplanes* sp. strain L3-i22 is 12,014,766 bp long, with a GC content of 71.4%, and the genome contains no plasmids. Based on the GTDB database, the taxonomic phylogeny of the assembled sequence was estimated, and the genome was best matched with Actinoplanes liguriensis (GenBank accession number GCA_000715855.1); however, the average nucleotide identity (ANI) value was 87.6%. Automatic annotation was conducted using an annotation pipeline, DFAST (13), provided by DDBJ, which predicted 10,740 coding sequences.

### Data availability.

The closed complete chromosomal sequence was deposited in DDBJ/EMBL/GenBank under accession number AP024745. Raw sequencing data were deposited in the DDBJ Sequence Read Archive (SRA) under accession numbers DRR299383 (DNBseq) and DRR299384 (GridION).
